# Developmental change in the association between adolescent depressive symptoms and the home environment: results from a longitudinal, genetically informative investigation

**DOI:** 10.1111/jcpp.12689

**Published:** 2017-02-02

**Authors:** Laurie J. Hannigan, Tom A. McAdams, Thalia C. Eley

**Affiliations:** ^1^ Institute of Psychiatry, Psychology & NeuroscienceMRC Social, Genetic & Developmental Psychiatry CentreKing's College LondonLondonUK

**Keywords:** Depression, adolescence, home environment, parenting, gene–environment correlation

## Abstract

**Background:**

Depression is already highly prevalent by late adolescence, indicating that research into its developmental emergence should consider earlier risk factors and environmental contexts. The home environment is a key context for children and adolescents throughout development. However, the nature of relationships that exist between aspects of the home environment and the development of depressive symptoms cannot be assumed. Genetically informative studies have been used to provide insights about the aetiology of such relationships, often finding them to be partly confounded by the influence of children's genes. Here, we investigate developmental change in the aetiology of the association between aspects of the home environment and depressive symptoms at the onset of adolescence.

**Methods:**

We used longitudinal child‐ and parent‐report data from >5,000 twin pairs enrolled in the UK‐representative Twins Early Development Study. Multivariate, genetically sensitive structural equation models were used to decompose latent variance and covariance in depressive symptoms (measured at 12 and 16 years) and aspects of the home environment (at 9 and 14 years) into genetic and environmental influences.

**Results:**

Going from childhood to adolescence, genetic influences accounted for an increasing proportion of the association [30% (16–42) of *r *=* *.44 in childhood; 40% (25–61) of *r *=* *.43 in adolescence], at the expense of shared environmental influences, which decreased from 70% (58–83) to 48% (29–62). Unique environmental influences accounted for a significant proportion of the association in adolescence only [12% (06–18)]. Developmental changes could largely be attributed to subtle shifts in the relative importance of stable aetiological factors, rather than the emergence of influences unique to adolescence.

**Conclusions:**

These findings emphasise the importance of developmental and aetiological context in interpreting associations between aspects of the home environment and child emotional outcomes.

## Introduction

Depression represents a substantial individual and societal burden across the life span (Costello, Mustillo, Erkanli, Keeler, & Angold, 2003; Merikangas et al., 2010). While the age‐of‐onset can vary widely, clinical depression is common in adolescence, with cumulative prevalence rates estimated at up to 10% by age 16 (Costello et al., 2003) and up to 20% by age 18 (Hankin et al., 1998; see Thapar, Collishaw, Pine, & Thapar, 2012 for a recent review). Adolescent depression is predictive of a range of future difficulties (Harrington, Fudge, Rutter, Pickles, & Hill, 1990, 1991; Harrington et al., 1994) and is itself predicted by earlier, more generalised emotional difficulties and symptoms (Birmaher et al., 1996). In part, this developmental continuity of risk seems to reflect a stable, underlying genetic liability to the disorder in childhood/adolescence (Hannigan, Walaker, Waszczuk, McAdams, & Eley, 2017) and beyond (Nivard et al., 2015). However, environmental factors are also known to be important. The sharp increase in prevalence of depression and depressive symptoms with the onset of adolescence makes this period a significant one at which to investigate potential environmental risk factors.

One specific context in which environmental risk factors for the developmental emergence of psychopathology have traditionally been sought is the home environment. Defined here as the constellation of factors that shape an individual's experience within their household (including parenting, sibling interactions, physical characteristics of the home, and so on), the home environment is an important context for developmental research (Bronfenbrenner, 1986; Sameroff, 1986). This is because of its relatively constant presence across development, usually from infancy until late adolescence, and widespread associations with child and adolescent outcomes (Bradley, Corwyn, Burchinal, McAdoo, & Garcia Coll, 2001; Rutter, 1985a, 1985b). Adolescent depression and depressive symptoms, specifically, have been shown to be related to a range of measures of family functioning, with parent characteristics and parenting behaviours a particular focus for research (see reviews/meta‐analyses by McLeod, Weisz, & Wood, 2007; Sheeber, Hops, & Davis, 2001; Yap & Jorm, 2015).

Associations between aspects of the home environment and adolescent depression may reflect the effects of causal, environmental risk processes. However, this is not the only possible explanation. The home environment is shaped, in large part, by the behaviours and interactions of biologically related individuals (i.e. children, their parents and their siblings). Insofar as these behaviours and interactions are influenced by genetic predispositions, the nature of the home environment will be related to genes that are shared, to varying degrees, between different members of the family. Crucially, for an individual child within a household, this means that associations between their own behaviours (including symptoms of psychopathology) and aspects of their home environment may be *genetically confounded*. The term given to the processes by which this can occur is *gene–environment correlation* (*r*GE; Eley, Napolitano, Lau, & Gregory, 2010; Plomin, DeFries, & Loehlin, 1977; Scarr & McCartney, 1983). When aspects of a child's home environment are influenced by parents or siblings (with whom they share genes), then *passive rGE* can occur, wherein the child's environment is *passively* correlated with their genes (Plomin et al., 1977). When the child's own genetically influenced behaviour *evokes* changes in their home environment, this is known as *evocative r*GE. A correlation between genotype and environment that arises from the genetically influenced ‘seeking out’ of specific environments is *active r*GE – although the scope for this, as relevant to emotional development, is somewhat limited within the context of the home environment (Neiderhiser et al., 2004).

Gene–environment correlation, in all its forms, can confound associations between aspects of the home environment and child outcomes. For example, genetically informed studies of adolescent depression and the home environment have found that genes account for a portion of the phenotypic overlap between them (e.g. with family connectedness: Jacobson & Rowe, 1999; punitive discipline: Lau, Rijsdijk, & Eley, 2006; parent–child hostility: Lewis, Collishaw, Thapar, & Harold, 2014; parental and sibling negativity: Pike, McGuire, Hetherington, Reiss, & Plomin, 1996; parenting style and household chaos: Wilkinson, Trzaskowski, Haworth, & Eley, 2013). However, with the emergence of depressive symptoms in development an essentially dynamic process, establishing the extent of confounding in these associations using cross‐sectional studies alone may not be sufficient. Despite the mounting evidence implicating genetic factors in associations between depression and aspects of the home environment, the extent to which the aetiology of these associations changes *developmentally* has not been well explored.

Changes in the extent of *r*GE, relative to parental influence and other environmental factors, has been suggested as an explanation for depression being more heritable in adolescence than childhood (Eley & Stevenson, 1999; Rice, 2009; Scourfield et al., 2003; Silberg et al., 1999; Thapar & McGuffin, 1994; see Rice, 2010 for a review). Furthermore, increasing genetic influence on associations between depressive symptoms and behaviour‐dependent life events has been shown in adolescence (Rice, Harold, & Thapar, 2003; Silberg et al., 1999). However, only three previous studies have examined the aetiology of the association between adolescent depression and aspects of the home environment developmentally. The first found that genetic factors primarily explained the modest associations between parental negativity in early adolescence and child depressive symptoms later in adolescence (Neiderhiser, Reiss, Hetherington, & Plomin, 1999). Recent work on twins and adopted siblings at age 15 and 18 found a similar pattern of results in the mother–child (but not father–child) relationship (Samek et al., 2016). One further study, using a monozygotic twin differences design, found suggestive evidence of a familial association between adolescent depressive symptoms and perceived maternal support at age 14, independent of earlier (age 13) links, which is consistent with increasing evocative *r*GE underpinned by developmentally emerging genetic influences (Guimond et al., 2016). To our knowledge, no previous study has examined the aetiology of the overlap between depressive symptoms and the home environment longitudinally across the transition from childhood into adolescence.

### Aims of current study

The main aim of this study is to investigate developmental change in the nature of the relationship between depressive symptoms and aspects of the home environment. To do this, we use a longitudinal, child‐based twin design, wherein data are collected from pairs of twins and their parents. We examine the aetiology of the association between a general index of maladaptive processes in the home environment (informed by measures of parenting and household chaos) and depressive symptoms. In a child‐based genetic design, genetic influence on measures of the home environment can be interpreted as indicating active/evocative *r*GE (Avinun & Knafo, 2014; Neiderhiser et al., 2004). This is because *passive r*GE increases the similarity of identical and nonidentical twins’ home environments equally, and is thus modelled as a shared environmental influence. Accordingly, we can look at developmental *changes* in the aetiology of the association between the home environment and depressive symptoms for an indication of how the role of active/evocative *r*GE evolves, relative to other influences, in adolescence. In addition, we can assess whether developmental changes in the association are due to aetiological factors emerging during adolescence (as indicated in Guimond et al., 2016), or the continuing influence of factors present earlier in development.

## Methods

### Participants

Data were drawn from the Twins Early Development Study (TEDS), a population‐based study of twins born in England & Wales between 1994 and 1996 (Haworth, Davis, & Plomin, 2013). The original enrolment for TEDS involved 16,810 twin families, of which 13,694 provided data at first contact. The TEDS sample has remained broadly representative of the UK population through multiple waves of data collection (Haworth et al., 2013). All participants provided informed consent, and the project has ethical approval from the Institute of Psychiatry Ethics Committee.

The analysis sample for this study consisted of those families who provided data on parenting, household chaos and depressive symptoms at 9, 12, 14 and 16 years of age after exclusions (7% of original sample) for medical and other reasons (e.g. lack of zygosity information). The overall sample (*N* = 13,292) included all individuals who contributed data to the main analyses and was 48% male and 93% of white ethnicity. Monozygotic twins made up 35% of the sample. Of those who provided data at age 9, 81% also responded at age 12, 60% at age 14 and 62% at age 16. The results of tests for selective attrition are described in Results section.

### Measures

This study makes use of available data from the measures described below. Data were not available for every measure at each of the four measurement occasions; for clarity, the measurement occasions at which data were available for each measure are given in parentheses after the measure name.

*Parental discipline (9 and 14 years)* was assessed by parent and child responses on a four‐item measure adapted from the parenting domain of a semistructured interview (Deater‐Deckard, Dodge, Bates, & Pettit, 1998). Participants reported how often parents’ used various disciplinary strategies (smacking/slapping; telling off/shouting; explaining; being firm/calm) to deal with child misbehaviour. Higher overall scores reflected higher levels of ‘harsh parenting’. The internal consistency of this scale was quite low [Cronbach's alphas at 9 years: .42 (child); .44 (parent); 14 years: .46 (child); .44 (parent)].

*Parental feelings (9 and 14 years)* were assessed using parent and child responses to a seven‐item version of the Parental Feelings Questionnaire (Deater‐Deckard & O'Connor, 2000). Three positive items (e.g. ‘I feel happy about my relationship with my child’) were retained from the original, alongside four negative items (e.g. ‘My Mum/Dad gets impatient with me’). Higher scores reflected greater negativity from parents towards their children. The internal consistency of this scale was reasonable [Cronbach's alphas at 9 years: .63 (child); .68 (parent); 14 years: .75 (child); .68 (parent)].

*Household chaos (9 and 14 years)* was assessed using six items from the Confusion, Hubbub and Order Scale (CHAOS; Matheny, Wachs, Ludwig, & Phillips, 1995). Children were asked to respond ‘Certainly true’, ‘Somewhat true’ or ‘Not true’ to items such as ‘You can't hear yourself think in our home’. While a parent‐report version of this measure was administered, responses were necessarily invariant across members of a twin pair (i.e. the parent reported once on the home environment in general), so this version of the CHAOS scale could not be incorporated. Thus, more so than for any of the other variables, this measure can be considered an index of adolescents’ *perceptions* (of, in this case, household chaos). The internal consistency of this scale was moderate [Cronbach's alphas: .58 (9 years); .56 (14 years)].

The internal consistency of some of the scales used to index the home environment was somewhat lower than the typical standard for acceptability in psychometric measures. While the reasons for this and implications have been discussed elsewhere (Hannigan, McAdams, Plomin, & Eley, 2016; Oliver, Trzaskowski, & Plomin, 2013), the modelling strategy used here is designed to maximise reliable, common variance in the home environment (see ‘Genetic analyses’ below).

*Depressive symptoms (12 and 16 years)* in twins were measured using the Short Mood and Feelings Questionnaire, a 13‐item measure assessing how often depressive symptoms occurred in the past 2 weeks (sMFQ; Angold et al., 1995). Responses were summed and the resultant scale had good internal consistency [Cronbach's alphas at 12 years: .85 (child); .84 (parent); at 16 years: .86 (child); .86 (parent)].

### Genetic analyses

The twin design exploits differences in the genetic relatedness of monozygotic (MZ) and dizygotic (DZ) twin pairs to estimate the relative contributions of, typically, additive genetic (A), shared environmental (C) and unique environmental (E) factors to variance in a measured phenotype. While reared‐together twins of either zygosity share their childhood environment, MZ twins share twice as much of their segregating genetic material (100%) as do DZ twins (≈50%). To the extent that the phenotypic similarity of MZ twins exceeds that of DZ twins, this is interpreted as evidence for the influence of additive genetic factors. When this difference is less than the difference in genetic relatedness between the pairs, this is interpreted as the influence of the shared environment. Nongenetic factors that make twins different from one another, whose influence can be seen in the extent that MZ twin correlations are less than unity, are estimated as unique environmental influences.

Structural equation modelling can be used to decompose twin variance/covariance matrices and, based on the rationale above, derive estimates for the components of phenotypic variance (Neale & Cardon, 1992). Where data from multiple different phenotypes, measurement occasions or reporters are available, the same procedure can be applied to conduct multivariate genetic analyses. In these analyses, it is possible to estimate the influence of genetic, shared environmental and unique environmental factors not only on variance in one phenotype but also on the covariance between phenotypes.

**Figure 1:**
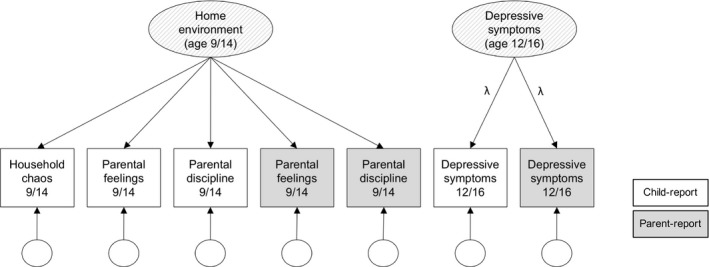
Path diagram of the ‘measurement’ component that formed the basis of all models, in which latent home environment and depressive symptoms phenotypes are estimated from observed variables. Note. Childhood and adolescent variables are collapsed here to save space, but separate latent variables are estimated for ages 9, 14 (home environment), 12 and 16 (depressive symptoms). Variance in observed variables is decomposed into common (in the latent phenotype) and residual variance. All factor loadings are freely estimated in the models; however, paths from the depressive symptoms latent variables (*λ*) are always equated to ensure model identification

The multivariate models applied to the data in this study consisted of a measurement component and a structural component. In the measurement component (Figure 1), latent variables loaded on the observed variables at each wave. This latent variable approach helped reduce bias and error in parameter estimates and was specified to best account for the structure and reliability of the available data. Specifically, the home environment latent variables extracted the most useful and reliable information from the observed variables, with freely estimated path loadings allowing each variable to contribute differentially to the latent construct, depending on reliability and relevance. The depressive symptoms latent variables reduced the influence of rater‐specific biases in the structural component of the model, with path loadings (*λ*) to parent and child reports equated. Residual variance specific to each of the 14 observed variables was decomposed separately.

**Figure 2:**
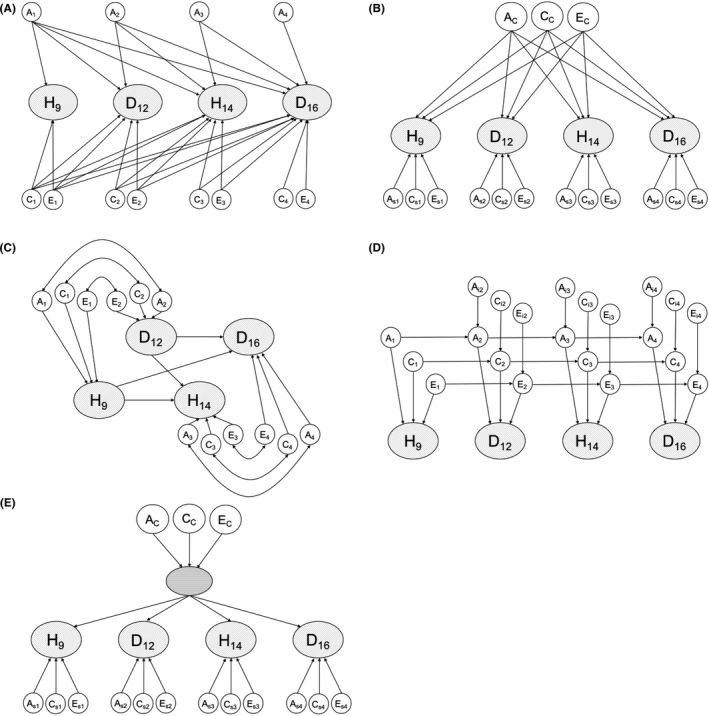
Specifications of structural components of genetic models used to explain latent variance/covariance in the home environment and depressive symptoms in late childhood and early/mid‐adolescence. Note. Observed variables and residual variance decomposition not shown. Models: A, Cholesky decomposition; B, independent pathway model; C, cross‐lag model; D, auto‐regressive simplex model; E, common pathway model; H, home environment; D, depressive symptoms; A, genetic factors; C, shared environmental factors; E, unique environmental factors

In the structural component of the models (Figure 2), variance and covariance in the latent factors were decomposed into correlated ‘A’, ‘C’ and ‘E’ aetiological components.

The structural component of the models was specified in five different ways to investigate the developmental structure of the data. In the Cholesky decomposition (panel A), variance components from earlier latent variables (ordered chronologically by age) explain variance at all subsequent waves. In the independent pathway model (panel B), temporally stable aetiological factors influence latent variables via individual paths, with age‐specific factors accounting for the remaining (latent) variance at each wave. In the cross‐lagged model (panel C), latent variables from adolescence (14 and 16) are regressed on those from childhood (9 and 12), and the variance/covariance of each pair of latent variables is decomposed separately. In the autoregressive simplex model (panel D), auto‐regressive paths link variance components with those immediately preceding them and may also be influenced by time‐specific innovations. Finally, in the common pathway model (panel E), temporally stable aetiological factors influence the latent variables via a single latent factor, which loads on them differentially, with age‐specific factors accounting for the remaining (latent) variance at each wave. Fit statistics from these specifications were compared, and parameter estimates from the best‐fitting model(s) were inspected to ascertain the extent and nature of developmental change in the aetiological relationship between the home environment and depressive symptoms.

The raw data were regressed on age and sex prior to analysis. In addition, variables with excessively skewed distributions (all depression variables) were log transformed. All models incorporated a scalar to account for variance differences between males and females. Model fitting was carried out in R using OpenMx v2.3.1 (Neale et al., 2015). OpenMx uses full‐information maximum likelihood for model parameter estimation.

## Results

Descriptive statistics are presented by sex and zygosity in Table S1, available online. Significant increases in mean levels of depressive symptoms between childhood (*M *=* *2.25, *SD *=* *3.37) and adolescence (*M *=* *3.40, *SD *=* *4.14) were observed for females’ self‐reports only [*t*(4,307)* *=* *16.54, *p *<* *0.001]. For all other variables, mean change over time was either nonsignificant or represented a slight but significant decrease (i.e. marginally lower levels of either depressive symptoms, household chaos, parental feelings or parental discipline) between childhood and adolescence. This was in line with findings of low‐level selective attrition indicated by slightly elevated mean scores in childhood for individuals who failed to provide data in adolescence, compared with those for whom adolescent data were available, on several variables (see Table S2 for details).

Phenotypic correlations between the observed variables are available in Table S3. Pairwise associations between individual observed home environmental and depressive symptoms variables ranged between .06 and .23. Within‐rater correlations typically exceeded cross‐rater correlations. On average, variables intercorrelated at around .30–.40 within measurement occasion.

### Genetic analyses

Fit statistics of the genetic models are presented in Table 1. Three specifications provided a comparable fit to the data. These were the Cholesky decomposition (−2LL = 258,717.70, AIC = 46,401.75), the independent pathway model (−2LL = 258,793.79, AIC = 46,465.79) and the cross‐lagged model (−2LL = 258,770.57, AIC = 46,438.57). No formal test is available to compare the fit statistics from nonnested models, and estimates from all three specifications were consistent with regard to broad patterns of aetiological stability and change and support the same overall conclusions. We have opted to present the results of the cross‐lagged model based on its parsimony, and because it is structured such that specific results germane to our research questions are conveniently delineated. For the interested reader, results from the Cholesky decomposition are also presented in Figure S1 and Table S4.

**Table 1:** Fit statistics from the genetic modelling of children's home environments and depressive symptoms in late childhood and early/mid‐adolescence

	ep	−2LL	*df*	AIC*df*
Cholesky decomposition	126	258,717.7	106,158	46,401.75
Independent pathway model	120	258,793.79	106,164	46,465.79
Cross‐lagged model	**118**	**258,770.57**	**106,166**	**46,438.57**
Autoregressive simplex model	117	259,048.28	106,167	46,714.28
Common pathway model	115	259,064.33	106,151	46,762.33

Best‐fitting developmental model in bold typeface. ep, estimated parameters; −2LL, minus 2 log‐likelihood fit index; *df*, degrees of freedom (actual); AIC*df*, Akaike's information criterion with *df* penalty.

As a preliminary test of the hypothesis that the aetiology of association between the home environment and depressive symptoms would be subject to developmental change, we tested versions of the models where the covariance structure of the data was constrained to be the same in childhood and adolescence. Imposing these constraints resulted in a significant and substantial worsening of fit in all cases. We, therefore, present and interpret parameter estimates from the full cross‐lagged model below.

**Figure 3:**
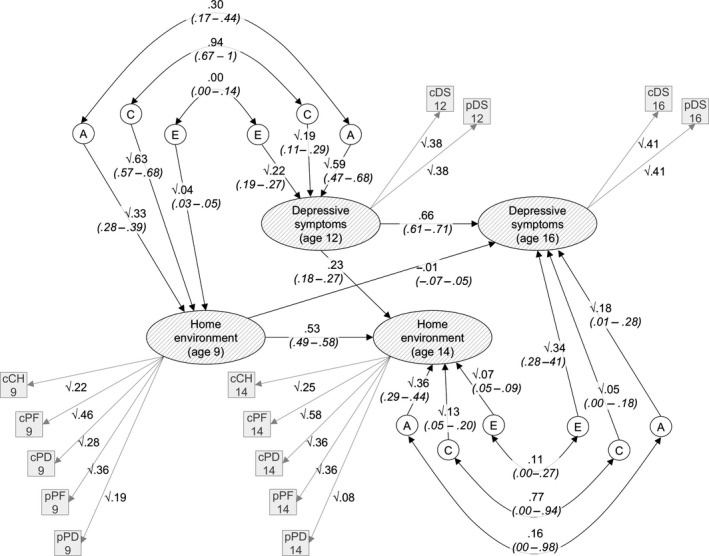
Estimates from the cross‐lagged model of the home environment and depressive symptoms in late childhood and early/mid‐adolescence. Note. Residual variance of observed variables estimated but not shown. Values on single headed arrows are path estimates; values on double‐headed arrows are correlation coefficients; 95% CIs given below each estimate in parentheses; A (genetic), C (shared environmental) and E (unique environmental) factors; c, child report; p, parent report; CH, household chaos; PD, parental discipline; PF, parental feelings; DZ, depressive symptoms

Path estimates from the structural component of the cross‐lagged model are presented in Figure 3 (estimates from the decomposition of residual variance in the measurement component are not shown; these are available in Table S5). On average, approximately one third of the variance of the observed variables was accounted for by the latent variable structure of the model, as indicated by the squared factor loadings (i.e. 22% variance in child‐reported household chaos at 9 is accounted for by the childhood home environment factor, as compared with 46% variance in child‐reported parental feelings).

Estimates from the cross‐lagged model, as shown in Figure 3, summarise the variance/covariance structure of the data across childhood and adolescence. Variance in childhood latent variables (home environment at 9 and depressive symptoms at 12) is entirely accounted for by the A, C and E components estimated for each. Similarly, covariance between childhood home environment and depressive symptoms is entirely explained by these A, C and E components and the correlations between them (double‐headed arrows in the top‐left of Figure 3). For example, the genetic covariance between the latent variables in childhood is √.33 × .30 × √.59 = .13, which equates to 30% of the overall association between them. In contrast, variance/covariance in the adolescent latent variables (home environment at 14 and depressive symptoms at 16) can be explained by both the adolescence‐specific variance/covariance structure (bottom‐right of Figure 3), and the childhood variance/covariance structure, via the central auto‐regressive and cross‐lagged paths. So, again taking genetic covariance as an example, there are four possible routes by which the adolescent latent variables can covary genetically: via the adolescence‐specific variance/covariance structure (√.18 × .16 × √.36 = .04); via the auto‐regressive paths and childhood variance/covariance structure (.66 × √.59 × .30 × √.33 × .53 = .05); and via the auto‐regressive and cross‐lag paths (.66 × .59 × .23 = .09 and −.01 × .33 × .53 = .00).

**Figure 4:**
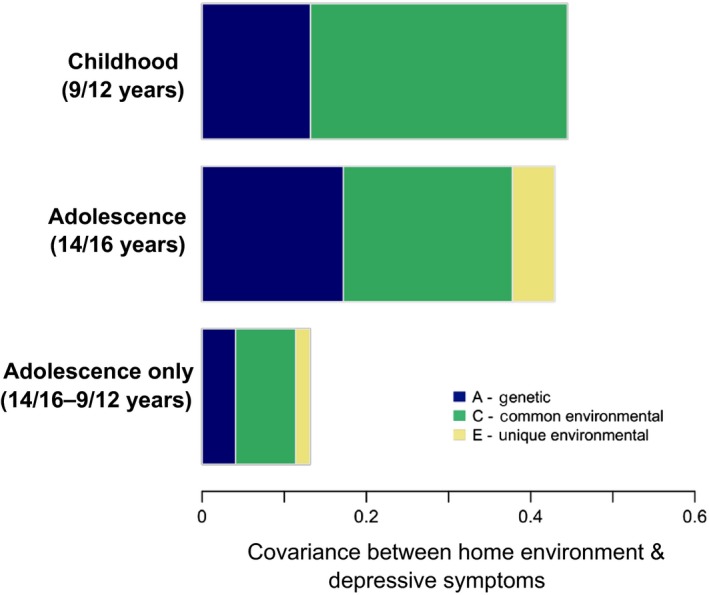
Decomposition of covariance between home environment and depressive symptoms factors in the cross‐lagged model [Colour figure can be viewed at wileyonlinelibrary.com]

The key information from this model, in terms of addressing the question of developmental change in the aetiology of the association between the home environment and depressive symptoms, is summarised in Figure 4. The overall length of the horizontal bars indicates the size of the phenotypic associations between the home environment and depressive symptoms factors. These associations were of similar magnitude in childhood [.44 (.40–.49)] and adolescence [.43 (.39–.47)]. Shaded areas of these bars represent the relative contributions of the correlated aetiological components to the covariance between the variables. In line with our prediction, changes in the aetiology of the association included an increase in the role of genetic factors in underpinning the association between the home environment and depressive symptoms (though confidence intervals overlapped) from accounting for 30% (16–42) of the covariation in childhood to 40% (25–61) in adolescence. This increase came at the expense of shared environmental factors, which went from explaining 70% (58–83) of the association in childhood to 48% (29–62). Unique environmental factors, which did not contribute to the association in childhood, explained the remaining 12% (06–18) in adolescence. However, after accounting for childhood aetiological influences and associations, less than one third of the phenotypic association in adolescence is attributable to influences unique to adolescence (lowest bar, Figure 4), with no significant contributions coming from adolescence‐specific factors. This indicates that the developmental changes observed are due to relative increases and decreases in the influence of aetiological factors present earlier in childhood.

## Discussion

In this study, we set out to investigate differences in the aetiology of the association between the home environment and depressive symptoms in adolescence versus childhood. Specifically, we aimed to establish whether an increase in confounding by genes in adolescence, shown previously for depression in relation to other environmental variables (Rice et al., 2003; Silberg et al., 1999), was a feature of the relationship between depressive symptoms and the home environment.

We observed both consistency and change in the aetiology of the association between the home environment and depressive symptoms. The magnitude of the association remained relatively constant between childhood and adolescence, and both genetic and shared environmental factors were involved significantly throughout. However, the role of genetic factors in explaining the association increased relative to shared environmental factors, and unique environmental factors also began to account for a proportion of the overlap in adolescence.

The shifting importance of different aetiological factors in the association between the home environment and depressive symptoms is informative as to potential changes in family processes at the onset of adolescence. Shared environmental influences incorporate all factors that make twins of either zygosity more similar to one another, including parental influence, neighbourhood characteristics and demographics such as socioeconomic status, among other possibilities (Avinun & Knafo, 2014; Burt, 2014). In adolescence, the relative importance of these shared environmental factors in the association decreased somewhat, though remaining predominant. These were partly replaced by the significant effects of unique environmental factors, indicating that the association between the home environment and depressive symptoms was partially explained, in adolescence, by factors that make twins of either zygosity different from one another. Specific unique environmental factors have typically proven difficult to identify (see Turkheimer & Waldron, 2000 for a review). However, shared environmental factors also gave way to increased confounding of the association by children's genes. The influence of children's genes on variance in their home environment can be interpreted as evidence of active/evocative *r*GE – that is, an association between children's genes and their environments via their behaviour (Avinun & Knafo, 2014; Neiderhiser et al., 2004). The home environment, modelled in our study as a latent index of child and parent reports on relevant questionnaires, was influenced by genes to a significantly greater extent in adolescence [52% (45–59)] compared with childhood [33% (28–39)]. This is consistent with the theoretical expectation that active/evocative *r*GE may increase as children age (Scarr & McCartney, 1983; see also: Marceau et al., 2016). Given this evidence for increasing active/evocative *r*GE within variance in the home environment, we interpret the increasing influence of child genes on the covariance with child depressive symptoms as also likely resulting from this increased active/evocative *r*GE.

Our results align with the findings of previous studies examining aetiological overlap between depressive symptoms and environmental factors in a developmental context (Guimond et al., 2016; Rice et al., 2003; Samek et al., 2016; Silberg et al., 1999). However, on the question of whether or not this increase could be attributed to genetic factors *unique* to adolescence, which has also been hypothesised (Rice, 2009), our results are less supportive. Instead, we find a pattern that is more similar to the ‘genetic amplification’ identified in a previous developmental study of the parent–child relationship in adolescence (Ludeke, Johnson, McGue, & Iacono, 2013), in which stable genetic influences become increasingly important relative to the declining effects of the factors comprising shared environmental influences. To our knowledge, our study is the first to show this pattern in covariance between measures of the home environment and depressive symptoms at the onset of adolescence.

### Limitations

This study is subject to the standard limitations and well‐explored assumptions of the classical twin design. However, several specific limitations should be highlighted. The first concerns the lack of availability of data on depressive symptoms and the home environment concurrently (i.e. all variables at all ages) and with equal intervals. The fact that we were unable to use data from parent reports on household chaos was also a limitation in terms of the balance of the observed variables. This was partially mitigated by equating factor loadings for parent and child reports of depressive symptoms, which reduced the effects of rater‐specific biases. Nonetheless, analyses of complete longitudinal data have the potential to further inform our understanding of developmental gene–environment interplay, not least because of the increasing range of sophisticated modelling strategies that may be applied to such data (Dolan, de Kort, van Beijsterveldt, Bartels, & Boomsma, 2014).

Finally, while we did make use of multiple measures to inform our ‘home environment’ factors, some of these had lower reliability coefficients than is desirable, while other key aspects of this context were not measured at all. Examples of this include variables like marital instability and information on sibling behaviours, both of which have been shown to correlate with depressive symptoms (Buist, Dekovic, & Prinzie, 2013; D'Onofrio et al., 2006). Aggregating information across the home environment more broadly could be an advantageous future direction.

## Conclusions

The results of this study are consistent with a picture of development‐related shifts in the aetiology of associations between variation in an individual's home environment and their depressive symptoms. Genetic confounding of these associations may increase in adolescence. However, as shown, any changes in the aetiology of associations can result from the amplification or attenuation of stable aetiological factors, rather than the influence of newly emerging factors. Developmental changes in an individual's capacity to shape their environment are an important consideration in the interpretation of associations between putative environments and developmental outcomes. Future research that aims to investigate the emergence of depression in adolescence should account for the nature of its associations with measured environments before drawing conclusions about the effects of an environmental exposure. The period of transition to adolescence remains fertile ground for exploring the aetiological origins of depression, while the ultimate goal of such work must always be to improve interventions and enhance clinical understanding.

**Key points**

- Associations between depression and measured aspects of the home environment have been frequently shown to be confounded by genetic influences, but developmental change in the nature of these associations during the transition from childhood to adolescence has not been well explored.
- We analysed data on the home environment and child depressive symptoms in middle childhood and adolescence using multivariate genetic models to compare the aetiology of the overlap across two periods of development.
- The aetiology of the association between the home environment and depressive symptoms changed in adolescence, via subtle increases in the roles of genetic and unique environmental factors and the declining influence of shared environmental factors.
- Changes primarily resulted from the amplification and attenuation of aetiological factors from childhood, rather than from the emergence of adolescence‐specific factors.
- Gene–environment correlation is a developmentally variable source of overlap between measures of the home environment and depressive symptoms, with potentially increasing importance in adolescence.

## Supporting information


**Table S1.** Descriptive statistics for raw data presented by sex and zygosity.
**Table S2.** Results of tests for selective attrition in the sample.
**Table S3.** Phenotypic correlations between observed variables (raw data) for males (below diagonal) and females (above diagonal) separately.
**Table S4.** Extent and composition of phenotypic associations between the home environment and depressive symptoms in childhood and adolescence from Cholesky decomposition.
**Table S5.** Path loadings from the measurement component of the genetic models (*λ*) and parameter estimates.
**Figure S1.** Estimates for variance components and factor loadings from Cholesky decomposition.

## References

[jcpp12689-bib-0001] Angold, A. , Costello, E.J. , Messer, S.C. , Pickles, A. , Winder, F. , & Silver, D. (1995). The development of a short questionnaire for use in epidemiological studies of depression in children and adolescents. International Journal of Methods in Psychiatric Research, 5, 237–249.

[jcpp12689-bib-0002] Avinun, R. , & Knafo, A. (2014). Parenting as a reaction evoked by children's genotype: A meta‐analysis of children‐as‐twins studies. Personality and Social Psychology Review, 18, 87–102. 23940232 10.1177/1088868313498308

[jcpp12689-bib-0003] Birmaher, B. , Ryan, N.D. , Williamson, D.E. , Brent, D.A. , Kaufman, J. , Dahl, R.E. , … & Nelson, B. (1996). Childhood and adolescent depression: A review of the past 10 years. Part I. Journal of the American Academy of Child and Adolescent Psychiatry, 35, 1427–1439. 8936909 10.1097/00004583-199611000-00011

[jcpp12689-bib-0004] Bradley, R.H. , Corwyn, R.F. , Burchinal, M. , McAdoo, H.P. , & Garcia Coll, C. (2001). The home environments of children in the United States part II: Relations with behavioral development through age thirteen. Child Development, 72, 1868–1886. 11768150 10.1111/1467-8624.t01-1-00383

[jcpp12689-bib-0005] Bronfenbrenner, U. (1986). Ecology of the family as a context for human development: Research perspectives. Developmental Psychology, 22, 723–742.

[jcpp12689-bib-0006] Buist, K.L. , Dekovic, M. , & Prinzie, P. (2013). Sibling relationship quality and psychopathology of children and adolescents: A meta‐analysis. Clinical Psychology Review, 33, 97–106. 23159327 10.1016/j.cpr.2012.10.007

[jcpp12689-bib-0007] Burt, S.A. (2014). Research Review: The shared environment as a key source of variability in child and adolescent psychopathology. Journal of Child Psychology and Psychiatry, 55, 304–312. 24261560 10.1111/jcpp.12173

[jcpp12689-bib-0008] Costello, E.J. , Mustillo, S. , Erkanli, A. , Keeler, G. , & Angold, A. (2003). Prevalence and development of psychiatric disorders in childhood and adolescence. Archives of General Psychiatry, 60, 837–844. 12912767 10.1001/archpsyc.60.8.837

[jcpp12689-bib-0009] Deater‐Deckard, K. , Dodge, K.A. , Bates, J.E. , & Pettit, G.S. (1998). Multiple risk factors in the development of externalizing behavior problems: Group and individual differences. Development and Psychopathology, 10, 469–493. 9741678 10.1017/s0954579498001709 PMC2776047

[jcpp12689-bib-0010] Deater‐Deckard, K. , & O'Connor, T.G. (2000). Parent‐child mutuality in early childhood: Two behavioral genetic studies. Developmental Psychology, 36, 561–570. 10976597 10.1037/0012-1649.36.5.561

[jcpp12689-bib-0011] Dolan, C.V. , de Kort, J.M. , van Beijsterveldt, T.C.E.M. , Bartels, M. , & Boomsma, D.I. (2014). GE covariance through phenotype to environment transmission: An assessment in longitudinal twin data and application to childhood anxiety. Behavior Genetics, 44, 240–253. 24789102 10.1007/s10519-014-9659-5 PMC4023080

[jcpp12689-bib-0012] D'Onofrio, B. , Turkheimer, E.N. , Emery, R.E. , Heath, A.C. , Madden, P. , Slutske, W.S. , & Martin, N. (2006). A genetically informed study of the processes underlying the association between parental marital instability and offspring adjustment. Developmental Psychology, 42, 486–499. 16756440 10.1037/0012-1649.42.3.486 PMC2965635

[jcpp12689-bib-0013] Eley, T. , Napolitano, M. , Lau, J.Y.F. , & Gregory, A.M. (2010). Does childhood anxiety evoke maternal control? A genetically informed study. Journal of Child Psychology and Psychiatry, 51, 772–779. 20202040 10.1111/j.1469-7610.2010.02227.x

[jcpp12689-bib-0014] Eley, T. , & Stevenson, J. (1999). Exploring the covariation between anxiety and depression symptoms: A genetic analysis of the effects of age and sex. Journal of Child Psychology and Psychiatry, 40, 1273–1282. 10604405

[jcpp12689-bib-0015] Guimond, F.‐A. , Laursen, B. , Vitaro, F. , Brendgen, M. , Dionne, G. , & Boivin, M. (2016). Associations between mother‐child relationship quality and adolescent adjustment: Using a genetically controlled design to determine the direction and magnitude of effects. International Journal of Behavioral Development, 40, 196–204. 27340309 10.1177/0165025415620059 PMC4914139

[jcpp12689-bib-0016] Hankin, B.L. , Abramson, L.Y. , Moffitt, T.E. , Silva, P.A. , McGee, R. , & Angell, K.E. (1998). Development of depression from preadolescence to young adulthood: Emerging gender differences in a 10‐year longitudinal study. Journal of Abnormal Psychology, 107, 128–140. 9505045 10.1037//0021-843x.107.1.128

[jcpp12689-bib-0017] Hannigan, L.J. , McAdams, T.A. , Plomin, R. , & Eley, T.C. (2016). Etiological influences on perceptions of parenting: A longitudinal, multi‐informant twin study. Journal of Youth and Adolescence, 45, 2387–2405. 26815663 10.1007/s10964-016-0419-0 PMC5101284

[jcpp12689-bib-0018] Hannigan, L.J. , Walaker, N. , Waszczuk, M.A. , McAdams, T. , & Eley, T. (2017). Aetiological influences on stability and change in emotional and behavioural problems across development: A systematic review. Psychopathology Review, 4, 52–108. 28337341 10.5127/pr.038315 PMC5360234

[jcpp12689-bib-0019] Harrington, R. , Bredenkamp, D. , Groothues, C. , Rutter, M. , Fudge, H. , & Pickles, A. (1994). Adult outcomes of childhood and adolescent depression. III. Links with suicidal behaviours. Journal of Child Psychology and Psychiatry, 35, 1309–1319. 7806612 10.1111/j.1469-7610.1994.tb01236.x

[jcpp12689-bib-0020] Harrington, R. , Fudge, H. , Rutter, M. , Pickles, A. , & Hill, J. (1990). Adult outcomes of childhood and adolescent depression. I. Psychiatric status. Archives of General Psychiatry, 47, 465–473. 2184797 10.1001/archpsyc.1990.01810170065010

[jcpp12689-bib-0021] Harrington, R. , Fudge, H. , Rutter, M. , Pickles, A. , & Hill, J. (1991). Adult outcomes of childhood and adolescent depression: II. Links with antisocial disorders. Journal of the American Academy of Child and Adolescent Psychiatry, 30, 434–439. 2055880 10.1097/00004583-199105000-00013

[jcpp12689-bib-0022] Haworth, C.M.A. , Davis, O.S.P. , & Plomin, R. (2013). Twins Early Development Study (TEDS): A genetically sensitive investigation of cognitive and behavioral development from childhood to young adulthood. Twin Research and Human Genetics, 16, 117–125. 23110994 10.1017/thg.2012.91 PMC3817931

[jcpp12689-bib-0023] Jacobson, K.C. , & Rowe, D.C. (1999). Genetic and environmental influences on the relationships between family connectedness, school connectedness, and adolescent depressed mood: Sex differences. Developmental Psychology, 35, 926–939. 10442862 10.1037//0012-1649.35.4.926

[jcpp12689-bib-0024] Lau, J.Y.F. , Rijsdijk, F.V. , & Eley, T. (2006). I think, therefore I am: A twin study of attributional style in adolescents. Journal of Child Psychology and Psychiatry, 47, 696–703. 16790004 10.1111/j.1469-7610.2005.01532.x

[jcpp12689-bib-0025] Lewis, G. , Collishaw, S. , Thapar, A. , & Harold, G.T. (2014). Parent‐child hostility and child and adolescent depression symptoms: The direction of effects, role of genetic factors and gender. European Child and Adolescent Psychiatry, 23, 317–327. 23963643 10.1007/s00787-013-0460-4

[jcpp12689-bib-0026] Ludeke, S. , Johnson, W. , McGue, M. , & Iacono, W.G. (2013). Genetic amplification and the individualization of the parent‐child relationship across adolescence. Psychological Medicine, 43, 413–422. 22874583 10.1017/S0033291712001201 PMC3495089

[jcpp12689-bib-0027] Marceau, K. , Knopik, V.S. , Neiderhiser, J.M. , Lichtenstein, P. , Spotts, E.L. , Ganiban, J.M. , & Reiss, D. (2016). Adolescent age moderates genetic and environmental influences on parent–adolescent positivity and negativity: Implications for genotype–environment correlation. Development and Psychopathology, 28, 149–166. 25924807 10.1017/S0954579415000358 PMC4627902

[jcpp12689-bib-0028] Matheny Jr, A. , Wachs, T. , Ludwig, J. , & Phillips, K. (1995). Bringing order out of chaos: Psychometric characteristics of the confusion, hubbub, and order scale. Journal of Applied Developmental Psychology, 16, 429–444.

[jcpp12689-bib-0029] McLeod, B.D. , Weisz, J.R. , & Wood, J.J. (2007). Examining the association between parenting and childhood depression: A meta‐analysis. Clinical Psychology Review, 27, 986–1003. 17449154 10.1016/j.cpr.2007.03.001

[jcpp12689-bib-0030] Merikangas, K.R. , He, J.‐P. , Burstein, M. , Swanson, S.A. , Avenevoli, S. , Cui, L. , … & Swendsen, J. (2010). Lifetime prevalence of mental disorders in U.S. adolescents: Results from the National Comorbidity Survey Replication‐Adolescent Supplement (NCS‐A). Journal of the American Academy of Child and Adolescent Psychiatry, 49, 980–989. 20855043 10.1016/j.jaac.2010.05.017 PMC2946114

[jcpp12689-bib-0031] Neale, M.C. , & Cardon, L.R. (1992). Methodology for genetic studies of twins and families. Dordrecht, the Netherlands: Springer, Netherlands.

[jcpp12689-bib-0032] Neale, M.C. , Hunter, M.D. , Pritikin, J.N. , Zahery, M. , Brick, T.R. , Kirkpatrick, R.M. , … & Boker, S.M. (2015). OpenMx 2.0: Extended structural equation and statistical modeling. Psychometrika, 26, 99–127. 10.1007/s11336-014-9435-8 PMC4516707 25622929

[jcpp12689-bib-0033] Neiderhiser, J.M. , Reiss, D. , Hetherington, E.M. , & Plomin, R. (1999). Relationships between parenting and adolescent adjustment over time: Genetic and environmental contributions. Developmental Psychology, 35, 680–692. 10380859 10.1037//0012-1649.35.3.680

[jcpp12689-bib-0034] Neiderhiser, J.M. , Reiss, D. , Pedersen, N.L. , Lichtenstein, P. , Spotts, E.L. , Hansson, K. , … & Ellhammer, O. (2004). Genetic and environmental influences on mothering of adolescents: A comparison of two samples. Developmental Psychology, 40, 335–351. 15122961 10.1037/0012-1649.40.3.335 PMC1226934

[jcpp12689-bib-0035] Nivard, M.G. , Dolan, C.V. , Kendler, K.S. , Kan, K.‐J. , Willemsen, G. , van Beijsterveldt, C.E.M. , … & Boomsma, D.I. (2015). Stability in symptoms of anxiety and depression as a function of genotype and environment: A longitudinal twin study from ages 3 to 63 years. Psychological Medicine, 45, 1039–1049. 25187475 10.1017/S003329171400213X

[jcpp12689-bib-0036] Oliver, B.R. , Trzaskowski, M. , & Plomin, R. (2013). Genetics of parenting: The power of the dark side. Developmental Psychology, 50, 1233–1240. 24364831 10.1037/a0035388 PMC3977675

[jcpp12689-bib-0037] Pike, A. , McGuire, S. , Hetherington, E.M. , Reiss, D. , & Plomin, R. (1996). Family environment and adolescent depressive symptoms and antisocial behavior: A multivariate genetic analysis. Developmental Psychology, 32, 590–603.

[jcpp12689-bib-0038] Plomin, R. , DeFries, J.C. , & Loehlin, J.C. (1977). Genotype‐environment interaction and correlation in the analysis of human behavior. Psychological Bulletin, 84, 309–322. 557211

[jcpp12689-bib-0039] Rice, F. (2009). The genetics of depression in childhood and adolescence. Current Psychiatry Reports, 11, 167–173. 19302772 10.1007/s11920-009-0026-9

[jcpp12689-bib-0100] Rice, F. (2010). Genetics of childhood and adolescent depression: Insights into etiological heterogeneity and challenges for future genomic research. Genome Medicine, 2, 68. 20860851 10.1186/gm189 PMC3092119

[jcpp12689-bib-0040] Rice, F. , Harold, G.T. , & Thapar, A. (2003). Negative life events as an account of age‐related differences in the genetic aetiology of depression in childhood and adolescence. Journal of Child Psychology and Psychiatry , 44, 977–987. 14531580 10.1111/1469-7610.00182

[jcpp12689-bib-0041] Rutter, M. (1985a). Family and school influences on behavioural development. Journal of Child Psychology and Psychiatry, 26, 349–368. 4008541 10.1111/j.1469-7610.1985.tb01938.x

[jcpp12689-bib-0042] Rutter, M. (1985b). Family and school influences on cognitive development. Journal of Child Psychology and Psychiatry, 26, 683–704. 3900115 10.1111/j.1469-7610.1985.tb00584.x

[jcpp12689-bib-0043] Samek, D.R. , Wilson, S. , Mcgue, M. , Iacono, W.G. , Samek, D.R. , Wilson, S. , … & Samek, D.R. (2016). Genetic and environmental influences on parent – Child conflict and child depression through late adolescence. Journal of Clinical Child and Adolescent Psychology, 1–6. 10.1080/15374416.2016.1141357 PMC5065391 27043719

[jcpp12689-bib-0044] Sameroff, A.J. (1986). Environmental context of child development. The Journal of Pediatrics, 109, 192–200. 3522835 10.1016/s0022-3476(86)80604-7

[jcpp12689-bib-0045] Scarr, S. , & McCartney, K. (1983). How people make their own environments: A theory of genotype greater than environment effects. Child Development, 54, 424–435. 6683622 10.1111/j.1467-8624.1983.tb03884.x

[jcpp12689-bib-0046] Scourfield, J. , Rice, F. , Thapar, A. , Harold, G.T. , Martin, N. , & McGuffin, P. (2003). Depressive symptoms in children and adolescents: Changing aetiological influences with development. Journal of Child Psychology and Psychiatry, 44, 968–976. 14531579 10.1111/1469-7610.00181

[jcpp12689-bib-0047] Sheeber, L. , Hops, H. , & Davis, B. (2001). Family processes in adolescent depression. Clinical Child and Family Psychology Review, 4, 19–35. 11388562 10.1023/a:1009524626436

[jcpp12689-bib-0048] Silberg, J.L. , Pickles, A. , Rutter, M. , Hewitt, J. , Simonoff, E. , Maes, H. , … & Eaves, L.J. (1999). The influence of genetic factors and life stress on depression among adolescent girls. Archives of General Psychiatry, 56, 225–232. 10078499 10.1001/archpsyc.56.3.225

[jcpp12689-bib-0049] Thapar, A. , Collishaw, S. , Pine, D.S. , & Thapar, A.K. (2012). Depression in adolescence. The Lancet, 379, 1056–1067. 10.1016/S0140-6736(11)60871-4 PMC3488279 22305766

[jcpp12689-bib-0050] Thapar, A. , & McGuffin, P. (1994). A twin study of depressive symptoms in childhood. British Journal of Psychiatry, 164, 259–265. 10.1192/bjp.165.2.259 7953041

[jcpp12689-bib-0051] Turkheimer, E.N. , & Waldron, M. (2000). Nonshared environment: A theoretical, methodological, and quantitative review. Psychological Bulletin, 126, 78–108. 10668351 10.1037/0033-2909.126.1.78

[jcpp12689-bib-0052] Wilkinson, P.O. , Trzaskowski, M. , Haworth, C.M.A. , & Eley, T. (2013). The role of gene‐environment correlations and interactions in middle childhood depressive symptoms. Development and Psychopathology, 25, 93–104. 23398755 10.1017/S0954579412000922 PMC3819563

[jcpp12689-bib-0053] Yap, M.B.H. , & Jorm, A. (2015). Parental factors associated with childhood anxiety, depression, and internalizing problems: A systematic review and meta‐analysis. Journal of Affective Disorders, 175, 424–440. 25679197 10.1016/j.jad.2015.01.050

