# Supplementary material for: Developmental change in the association between adolescent depressive symptoms and the home environment: results from a longitudinal, genetically informative investigation
Source: J Child Psychol Psychiatry. 2017 Feb 2;58(7):787–97. doi: 10.1111/jcpp.12689 (PMC5484341; doi:10.1111/jcpp.12689)
Supplement: Supplementary file 1 — Table S1. Descriptive statistics for raw data presented by sex and zygosity. Table S2. Results of tests for selective attrition in the sample. Table S3. Phenotypic correlations between observed variables (raw data) for males (below diagonal) and females (above diagonal) separately. Table S4. Extent and composition of phenotypic associations between the home environment and depressive symptoms in childhood and adolescence from Cholesky decomposition. Table S5. Path loadings from the measurement component of the genetic models (λ) and parameter estimates. Figure S1. Estimates for variance components and factor loadings from Cholesky decomposition. [file JCPP-58-787-s001.docx]

**Supporting information for *Adolescent depression and the home environment* by Hannigan et al.**

**Table S1**. Descriptive statistics for raw data presented by sex and zygosity

*Note – MZM: monozygotic male twins; MZF: monozygotic female twins; DZM: dizygotic male twins; DZF: dizygotic female twins; SD: standard deviation; N: number of individuals*

**Table S2.** Results of tests for selective attrition in the sample

Note – darker shading indicates stronger correlation (Pearson’s r)

**Table S3.** Phenotypic correlations between observed variables (raw data) for males (below diagonal) and females (above diagonal) separately

*Note – Latent correlation structure estimated but not shown;*

*Values are squared, standardised variance components/factor loadings;*

*95% CIs below latent factor variance components in italics;*

*A (genetic), C (shared environmental) and E (unique environmental) factors;*

*c- : child-report; p- : parent-report; CHAOS: household chaos; DIS: parental discipline; DIS: parental discipline; PFEE: parental feelings; MFQ: depressive symptoms;*

**Figure S1.** Estimates for variance components and factor loadings from the multivariate genetic model of measured aspects of the home environment and depressive symptoms in late childhood and early/mid-adolescence

**Table S4.** Extent and composition of phenotypic associations between the home environment and depressive symptoms in childhood and adolescence from Cholesky decomposition

| **rPh** |  | Home env. (9) |  | Depr. (12) |  | Home env. (14) |
| --- | --- | --- | --- | --- | --- | --- |
|  |  |  |  |  |  |  |
| Depr. (12) |  | 0.45 |  |  |  |  |
|  |  | *0.40−0.49* |  |  |  |  |
| Home env. (14) |  | 0.64 |  | 0.48 |  |  |
|  |  | *0.60−0.68* |  | *0.44−0.52* |  |  |
| Depr. (16) |  | 0.27 |  | 0.66 |  | 0.43 |
|  |  | *0.22−0.32* |  | *0.62−0.70* |  | *0.39−0.47* |
|  |  |  |  |  |  |  |
| **% rPh due to A** | | | | | | |
| Depr. (12) |  | 0.22 |  |  |  |  |
|  |  | *0.08−0.38* |  |  |  |  |
| Home env. (14) |  | 0.40 |  | 0.41 |  |  |
|  |  | *0.31−0.48* |  | *0.11−0.72* |  |  |
| Depr. (16) |  | 0.41 |  | 0.63 |  | 0.42 |
|  |  | *0.25−0.56* |  | *0.49−0.77* |  | *0.25−0.66* |
|  |  |  |  |  |  |  |
| **% rPh due to C** | | | | | | |
| Depr. (12) |  | 0.74 |  |  |  |  |
|  |  | 0*.60−0.87* |  |  |  |  |
| Home env. (14) |  | 0.60 |  | 0.51 |  |  |
|  |  | 0*.52−0.68* |  | 0*.37−0.65* |  |  |
| Depr. (16) |  | 0.59 |  | 0.20 |  | 0.48 |
|  |  | *0.29−0.88* |  | *0.09−0.32* |  | *0.29−0.63* |
|  |  |  |  |  |  |  |
| **% rPh due to E** | | | | | | |
| Depr. (12) |  | 0.04 |  |  |  |  |
|  |  | *0.00−0.08* |  |  |  |  |
| Home env. (14) |  | 0.00 |  | 0.08 |  |  |
|  |  | *0.00−0.02* |  | *0.04−0.13* |  |  |
| Depr. (16) |  | 0.00 |  | 0.16 |  | 0.10 |
|  |  | *0.00−0.05* |  | *0.11−0.22* |  | *0.04−0.15* |
|  |  |  |  |  |  |  |

*Note – rPh: phenotypic correlation; A: genetic factors; C: shared environmental factors; E unique environmental factors*

**Table S5**. Path loadings from the measurement component of the genetic models (λ) and parameter estimates from the decomposition of residual variance in measures of the home environment and depressive symptoms during late childhood and early adolescence

| Wave |  | Reporter |  | Measure |  | λ |  | As | *95% CIs* | Cs |  | Es |  |
| --- | --- | --- | --- | --- | --- | --- | --- | --- | --- | --- | --- | --- | --- |
| Age 9 |  | Child |  | Household chaos |  | **0.47** |  | 0.18 | *.00 - .25* | 0.21 | *.00 - .27* | **0.34** | *.31 - .37* |
|  |  |  |  | Parental feelings |  | **0.68** |  | 0.05 | *.00 - .08* | 0.00 | *.00 - .00* | **0.45** | *.42 - .48* |
|  |  |  |  | Parental discipline |  | **0.53** |  | 0.12 | *.00 - .22* | 0.09 | *.00 - .16* | **0.49** | *.45 - .53* |
|  |  | Parent |  | Parental feelings |  | **0.60** |  | **0.40** | *.35 - .44* | **0.21** | *.16 - .26* | **0.08** | *.07 - .09* |
|  |  |  |  | Parental discipline |  | **0.44** |  | **0.12** | *.11 - .14* | **0.66** | *.62 - .70* | **0.02** | *.01 - .02* |
| Age 12 |  | Child |  | Depressive symptoms |  | **0.62** |  | 0.10 | *.00 - .17* | 0.07 | *.00 - .13* | **0.44** | *.41 - .48* |
|  |  | Parent |  | Depressive symptoms |  | **0.62** |  | 0.24 | *.00 - .30* | **0.16** | *.11 - .21* | **0.26** | *.23 - .26* |
| Age 14 |  | Child |  | Household chaos |  | **0.50** |  | 0.07 | *.00 - .16* | **0.25** | *.12 - .32* | **0.42** | *.39 - .46* |
|  |  |  |  | Parental feelings |  | **0.76** |  | 0.03 | *.00 - .06* | 0.00 | *.00 - .00* | **0.41** | *.38 - .48* |
|  |  |  |  | Parental discipline |  | **0.60** |  | 0.09 | *.00 - .19* | 0.08 | *.00 - .15* | **0.49** | *.45 - .54* |
|  |  | Parent |  | Parental feelings |  | **0.60** |  | **0.34** | *.30 - .34* | **0.23** | *.19 - .28* | **0.07** | *.07 - .08* |
|  |  |  |  | Parental discipline |  | **0.29** |  | **0.11** | *.10 - .12* | **0.69** | *.65 - .73* | **0.02** | *.02 - .02* |
| Age 16 |  | Child |  | Depressive symptoms |  | **0.64** |  | **0.20** | *.17 - .24* | 0.00 | *.00 - .00* | **0.50** | *.46 - .54* |
|  |  | Parent |  | Depressive symptoms |  | **0.64** |  | 0.31 | *.00 - .40* | **0.14** | *.07 - .20* | **0.29** | *.26 - .33* |

*Note – significant estimates (Cis do not overlap zero) in bold; As = Variable-specific genetic influences; Cs = Variable-specific shared environmental influences; Ex = Variable-specific unique environmental influences*
